# Supplementary material for: A subset of cortical areas exhibit adult-like functional network patterns in early childhood
Source: Dev Cogn Neurosci. 2025 Mar 12;73:101551. doi: 10.1016/j.dcn.2025.101551 (PMC11978374; doi:10.1016/j.dcn.2025.101551)
Supplement: Supplementary file 1 — Supplementary material [file mmc1.docx]

**Supplementary Materials**

**Supplementary Results**

**S1. Silhouette index in individual sessions**

We calculated the silhouette index in group-average data because individual sessions are noisy and have a relatively short acquisition time. However, our group level results were consistent with results obtained by calculating silhouette index in individual sessions (Supplementary Figures 3-4). Specifically, the average SI (-0.0054 ± 0.0714) was not different from zero in individual adult sessions (two-tailed t-test, p = 0.41) for adult (“Gordon”) networks. On the other hand, the average SI (-0.0859 ± 0.0318) was significantly smaller than zero in individual adult sessions for infant (“Kardan”) networks (two-tailed t-test, p<0.001). In addition, the average SI (-0.0636 ± 0.0492) was smaller than zero in individual infant sessions (two-tailed t-test, p < 0.001) for adult(“Gordon”) networks. On the other hand, the average SI (0.0492 ± 0.0472) was significantly greater than zero in individual adult sessions for infant (“Kardan”) networks (two-tailed t-test, p<0.001).

**S2. Silhouette index of adult networks in infant FC with the mean in all alternative networks**

By default, the silhouette index compares the current network to the best alternative network, which also depends on the quality of alternatives. However, other researchers have chosen to use a similar metric that compares the average within-network similarity to the average between-network similarity across all alternative networks, rather than just the best alternative (Ji et al., 2019). This approach tends to be less conservative and generally results in a higher silhouette index when calculated in this manner.

When the SI was calculated using the mean in all alternative networks rather than the mean of the best alternative network, they were still moderately correlated with the SI reported in the main results (Pearson’s r = 0.74, p <0.001). However, since the mean of similarity to all alternative networks (especially to the ones spatially distant from the area in question) would tend to be lower than the best alternative, the SI is positively shifted with almost all parcels having SI > 0 (Supplementary Figure 15).

**S3. Age effect on within-network (Gordon networks) FC is smaller in magnitude with our area subset than with all areas**

To test the hypothesis that our area subset has relatively stable within-network FC across chronological age in infants, we compared the age effect on within-network FC when the networks include only our area subset versus all areas. The age effect of within-network FC was quantified with a Spearman’s correlation ($\rho$). The significance of the difference between the correlation between chronological age and within-network FC in our area subset versus all areas is calculated with a Z-test on Fisher-Z-transformed r values.

We additionally examined the within-network FC in infants across chronological age. We computed within-network FC across age using full versus our area subset. For the eight networks that were partially retained, five networks demonstrated a significant correlation between within-network FC and age (*p* < 0.05, Spearman’s *ρ*): the within-network Aud, SMN hand and Vis networks were negatively correlated with age and the within-network FC in DAN and the FPN were positively correlated with age. The age effect was greater in magnitude with the full set of areas (Figure 3A) than with only the partially retained areas (Figure 3B) for the SMN hand network, although not significant when comparing the Fisher-Z-transformed *ρ* values (*Z* = 1.588, one-sided p = 0.056). Similar results were found for other networks, where the age effect was less negative for Aud, SMN hand and Vis networks, and less positive for DAN and FPN, but none of them had a significant (p < 0.05) Z-test. To examine the robustness of our result to the selection of data samples, we generated 1000 bootstrapped samples of the infant sessions. We found that the sign of the difference was consistent across bootstrap samples (i.e., on average the networks using our area subset was less correlated with age than all areas) (Figure 3C). The mean and 95% confidence interval for the bootstrap showed a mean difference in Fisher-Z-transformed *ρ* values for full versus subset was -0.1139 [-0.1721,0.0129] for Aud, -0.1386 [-0.1684, -0.0814] for SMN hand, 0.0020 [-0.0887, 0.0348] for Vis, -0.0205 [-0.0120, 0.1439] for DAN and 0.0089 [0.0071, 0.0511] for FPN (Figure 3C).

**S4. Group consistency and differential power of FC edges**

Prior studies suggested that it was possible to identify individuals using FC in infants from the BCP dataset (Hu et al., 2022; Kardan et al., 2022). To assess which FC edges (i.e. connections between a pair of areas) are more consistent across individuals versus distinct across individuals, we calculated the group consistency ($\phi)$ and differential power (DP) measures (Finn et al., 2015). We aim to describe the distribution of highly consistent edges and highly differentiating edges with respect to adult and infant network models. For this analysis, we only use the one session from each of the 115 unique subjects with at least 13.2 min low-motion data. Given two sets of connectivity [$X_{i}^{R1}$], [$X_{i}^{R2}$] obtained from the two resting scan windows (*R1* and *R2*) after z-score normalization, the edgewise product vector $\varphi_{i}$was computed as

$\varphi_{i} \left( e \right)= X_{i}^{R1}\left( e \right)*X_{i}^{R2}\left( e \right), e=1,\ldots,M (Equation 2)$

where *i* indexed the subject, *e* indexed the edge, and *M* indexed the total number of FC edges. The sum of $\varphi_{i}$ over all edges is the correlation between [$X_{i}^{R1}$], [$X_{i}^{R2}$]. The group consistency $\phi$ was computed as the mean of $\varphi_{i}$ across all subjects. We defined the edges with the top 10% $\phi$ values to be “highly consistent”.

Similarly, the edgewise product vector $\varphi_{ij}$was calculated between patterns from different subjects, for example:

$\varphi_{ij} \left( e \right)= X_{i}^{R1}\left( e \right)*X_{j}^{R2}\left( e \right), e=1,\ldots,M, i\neq j (Equation 3)$

$P_{i}\left( e \right)= P\left| \varphi_{ij} \left( e \right)>\varphi_{ii} \left( e \right) or \varphi_{ji} \left( e \right)>\varphi_{ii} \left( e \right) \right| \left( Equation 4 \right)$

$DP \left( e \right)=\sum_{i} \left\{ -ln(P_{i}\left( e \right)) \right\} \left( Equation 5 \right)$

We defined the edges with the top 10% DP values as “highly differentiating”.

Consistent with the findings in previous literature, we observed that a large percentage (~50%) of FC edges in the within-network blocks tend to be highly consistent. On the other hand, much fewer FC edges in between-network blocks (~6%) were highly consistent (Supplementary Figure 14; Supplementary Table 3). The sensorimotor networks especially had a large proportion of highly consistent within-network FC edges (Supplementary Table 4). Moreover, using adult networks defined by our area subset, the percentage of highly consistent edges within networks increased substantially for all eight partially retained networks (Supplementary Table 4), indicating that the adult network spanned by our area subset over-represented areas with highly consistent FC between them.

On the other hand, within-network blocks tend to have only a slightly larger percentage of highly differentiating FC edges (~15%) than between-network blocks (~10%) (Supplementary Table 5-6), with both increased and decreased proportion of highly differentiating edges when using our area subset instead of all areas.

References

Finn, E. S., Shen, X., Scheinost, D., Rosenberg, M. D., Huang, J., Chun, M. M., Papademetris, X., & Constable, R. T. (2015). Functional connectome fingerprinting: Identifying individuals using patterns of brain connectivity. *Nature Neuroscience*, *18*(11), Article 11. https://doi.org/10.1038/nn.4135

Hu, D., Wang, F., Zhang, H., Wu, Z., Zhou, Z., Li, G., Wang, L., Lin, W., Li, G., & Consortium, U. B. C. P. (2022). Existence of Functional Connectome Fingerprint during Infancy and Its Stability over Months. *Journal of Neuroscience*, *42*(3), 377–389. https://doi.org/10.1523/JNEUROSCI.0480-21.2021

Ji, J. L., Spronk, M., Kulkarni, K., Repovš, G., Anticevic, A., & Cole, M. W. (2019). Mapping the human brain’s cortical-subcortical functional network organization. *NeuroImage*, *185*, 35–57. https://doi.org/10.1016/j.neuroimage.2018.10.006

Kardan, O., Kaplan, S., Wheelock, M. D., Feczko, E., Day, T. K. M., Miranda-Domínguez, Ó., Meyer, D., Eggebrecht, A. T., Moore, L. A., Sung, S., Chamberlain, T. A., Earl, E., Snider, K., Graham, A., Berman, M. G., Uğurbil, K., Yacoub, E., Elison, J. T., Smyser, C. D., … Rosenberg, M. D. (2022). Resting-state functional connectivity identifies individuals and predicts age in 8-to-26-month-olds. *Developmental Cognitive Neuroscience*, *56*, 101123. https://doi.org/10.1016/j.dcn.2022.101123

**Supplementary Figure 1.** *Distribution of age and sex of individual infants in the BCP dataset.* A) The age time points of 181 infants ordered by sex. B) The count of number of individuals with 1-6 longitudinal points.


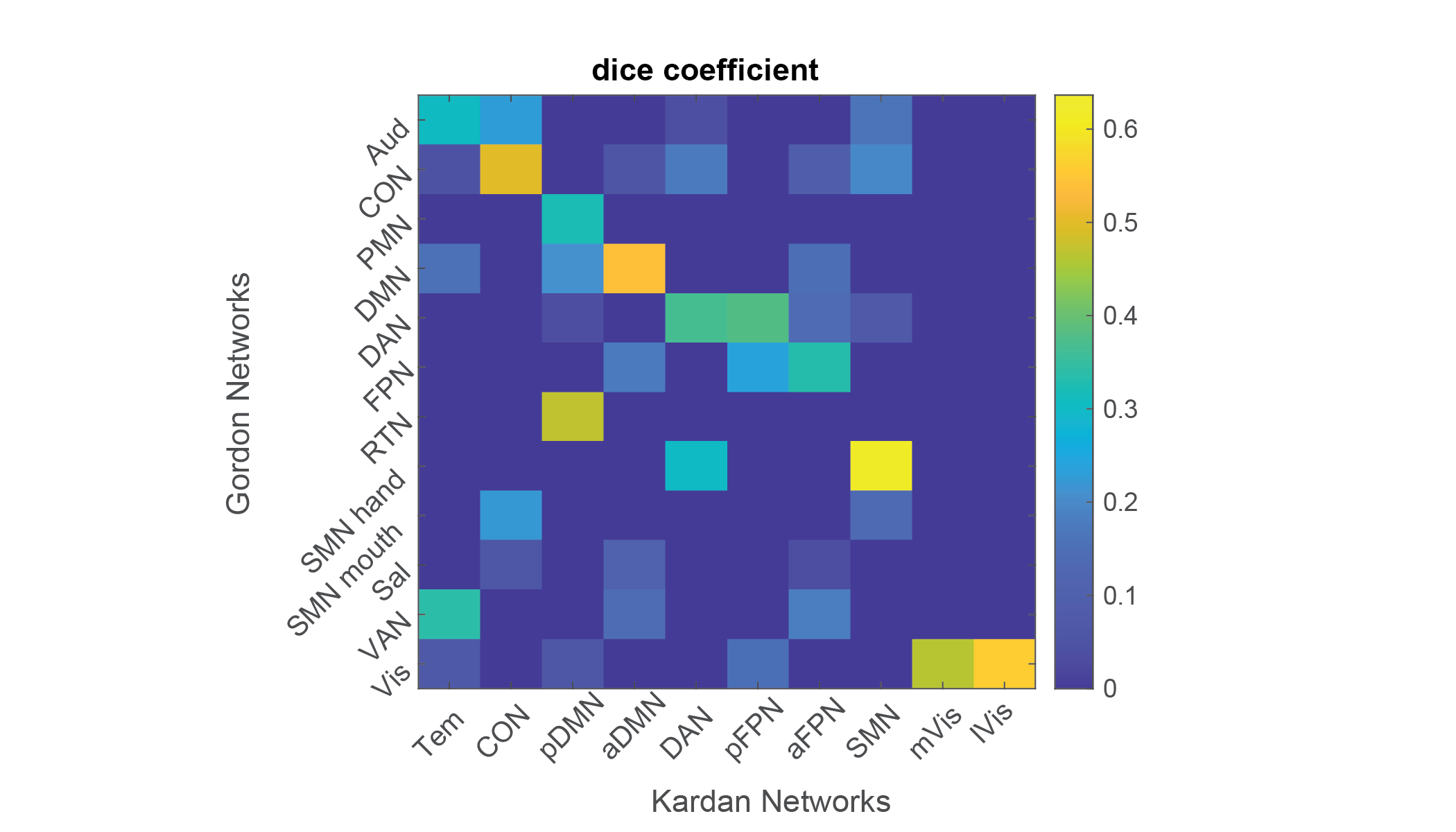


**Supplementary Figure 2.** *Dice overlap between Adult Networks (“Gordon”) and Infant Networks (“Kardan”).* Network abbreviations: auditory (Aud), cingulo-opercular (CON), parietal memory (PMN), default mode (DMN), dorsal attention (DAN), fronto-parietal (FPN), retrosplenial temporal (RTN), somatomotor hand (SMN hand), somatomotor mouth (SMN mouth), salience (Sal), and ventral attention (VAN), visual (Vis), somatomotor (SMN), temporal (Tem), posterior frontoparietal (pFPN), posterior default mode (pDMN), lateral visual (lVis), medial visual (mVis), anterior fronto-parietal (aFPN), anterior default mode (aDMN).


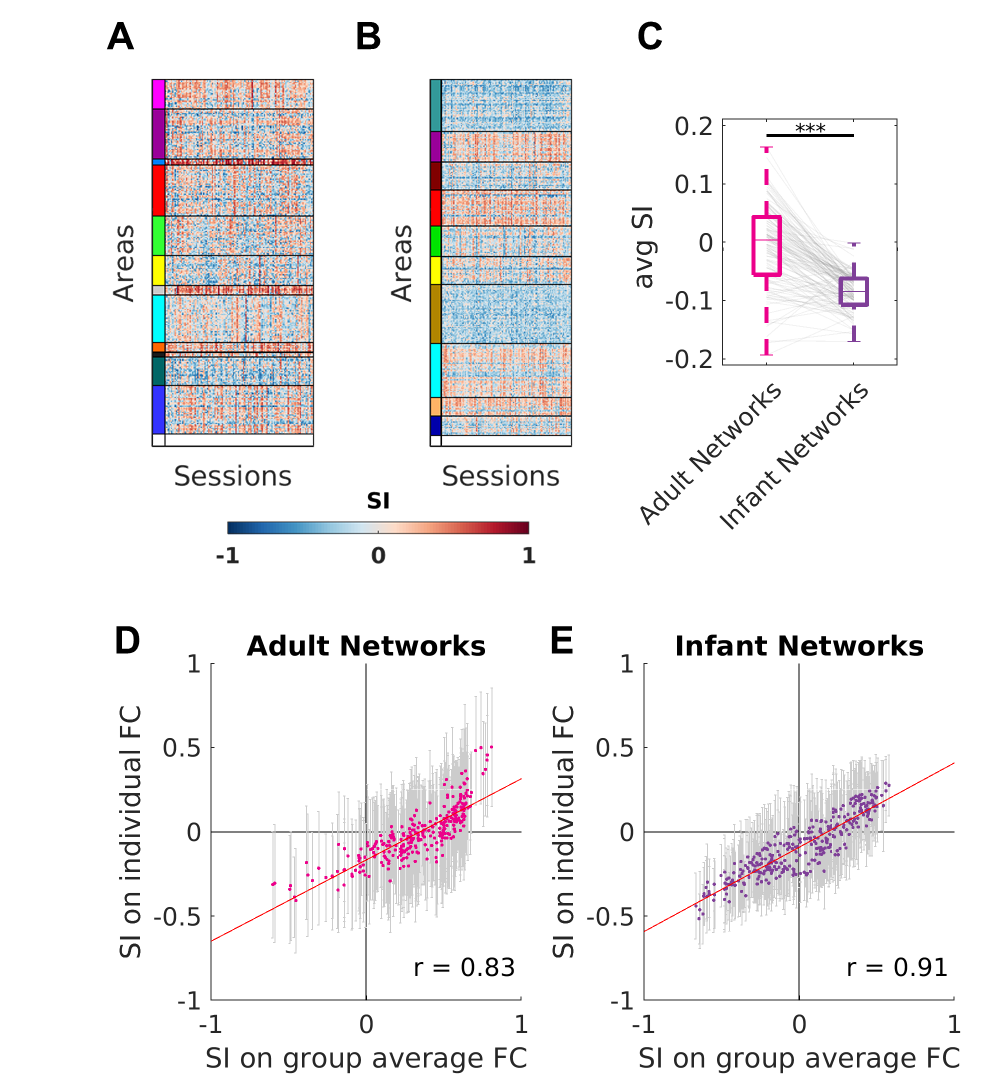


**Supplementary Figure 3.** *Silhouette index (SI) of adult and infant networks on individual adults’ FC.* A) SI across adult networks (“Gordon”, 286 areas). B) SI across infant networks (“Kardan”, 328 areas). C) average SI of adult and infant networks across areas on individual adults’ FC. *** p < 0.001 in paired t-test. D) Pearson’s correlation of SI of adult networks on group average FC and the mean of SI on individual FC across 286 areas. E) Pearson’s correlation of SI of infant networks on group average FC and the mean of SI on individual FC across 328 areas.


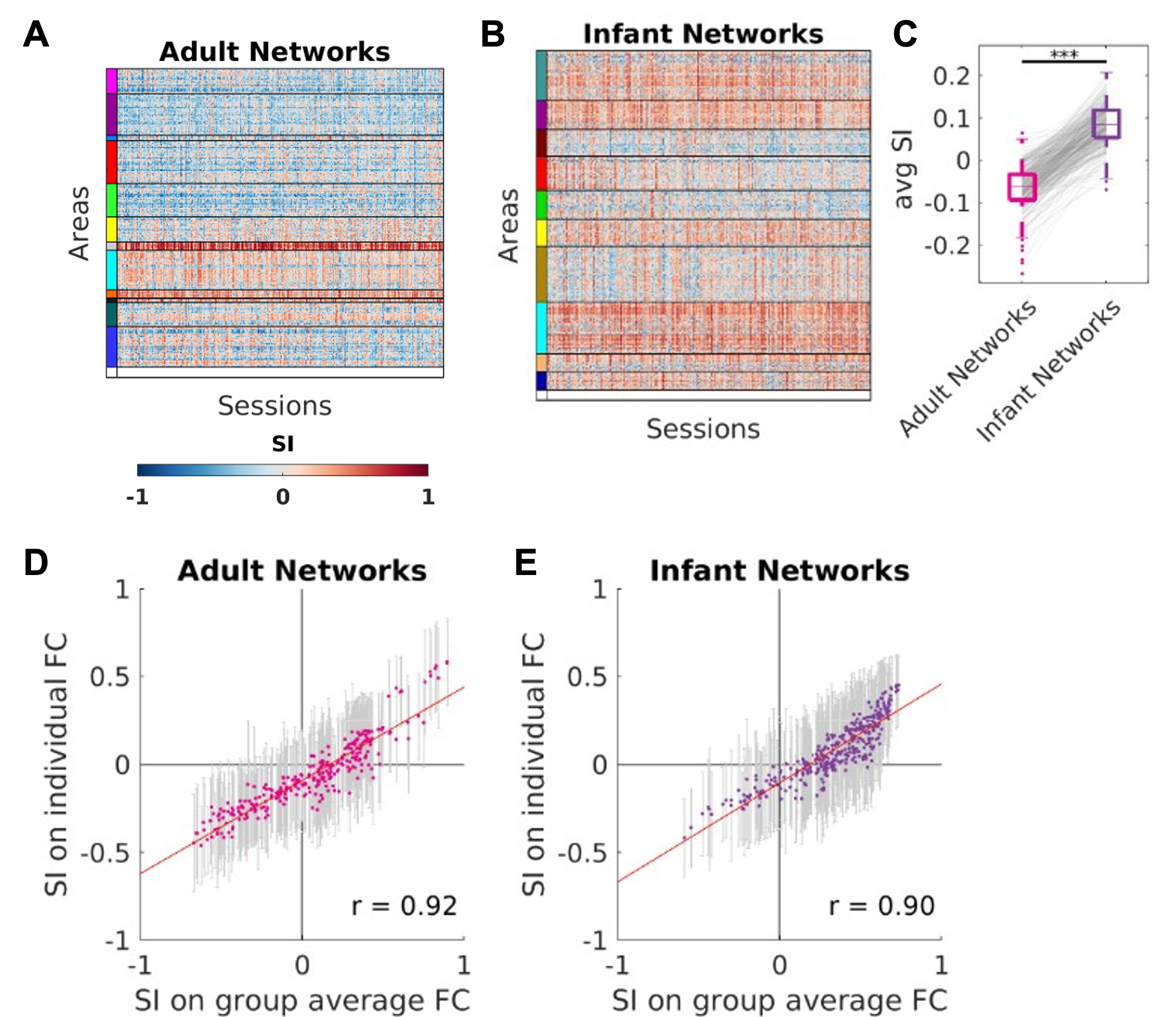


**Supplementary Figure 4.** *Silhouette index (SI) of adult and infant networks on individual infants’ FC.* A) SI across adult networks (“Gordon”, 286 areas). B) SI across infant networks (“Kardan”, 328 areas). C) average SI of adult and infant networks across areas on individual infants’ FC. *** p < 0.001 in paired t-test. D) Pearson’s correlation of SI of adult networks on group average FC and the mean of SI on individual FC across 286 areas. E) Pearson’s correlation of SI of infant networks on group average FC and the mean of SI on individual FC across 328 areas. Sessions in A and B are sorted by increasing age from left to right.


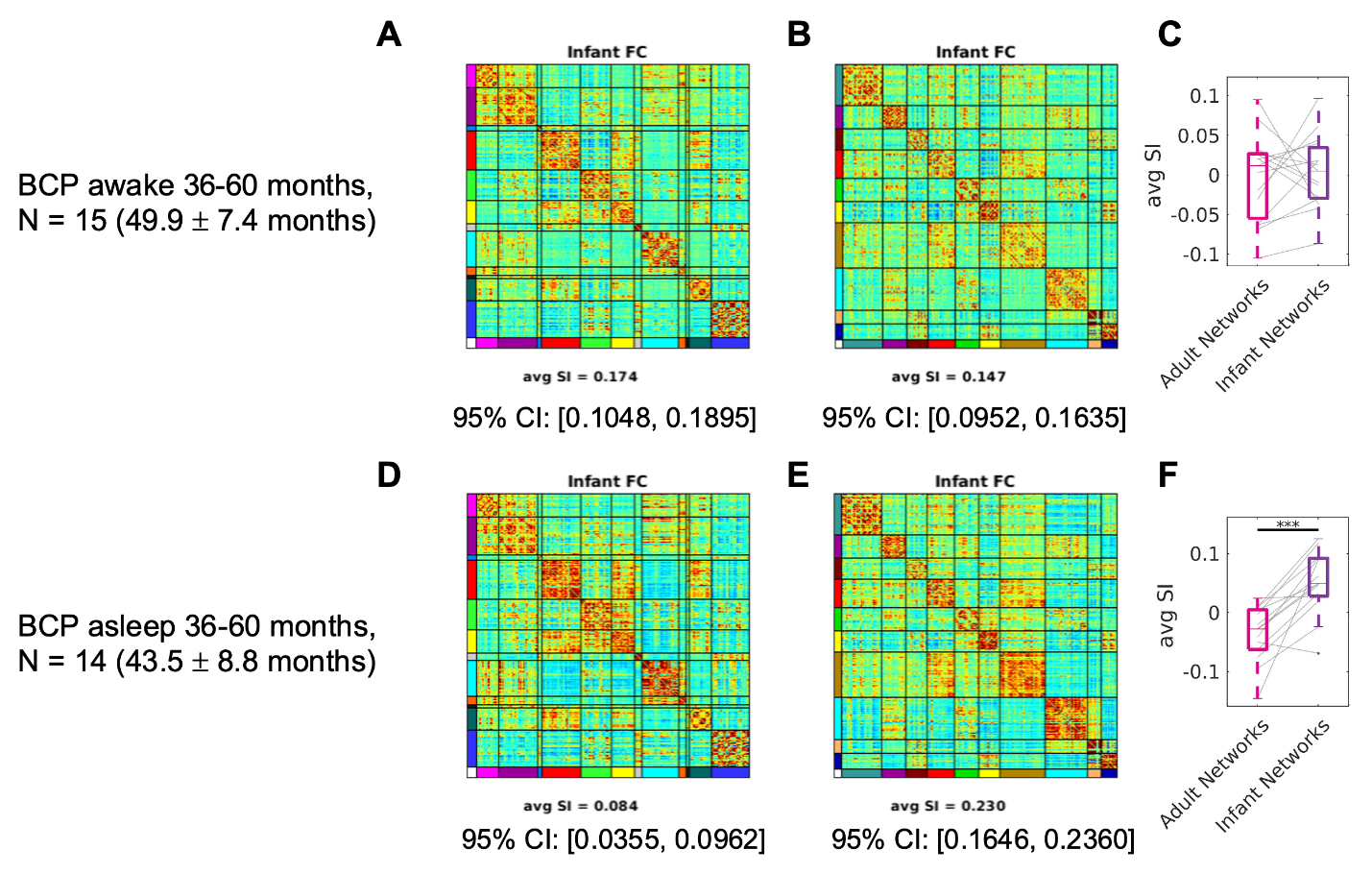


**Supplementary Figure 5.** *Awake V.S. sleeping infant FC organized by adult (“Gordon”) and infant (“Kardan”) networks.* A) The average FC for 15 BCP sessions sorted by adult networks (“Gordon”). B) The average FC for 15 BCP sessions sorted by infant networks (“Kardan”). C) Average silhouette index for individual sessions. D-F) Same as A-C, but for 14 BCP sessions also in approximately the same age range (36-60 months). *** *p* < 0.001.


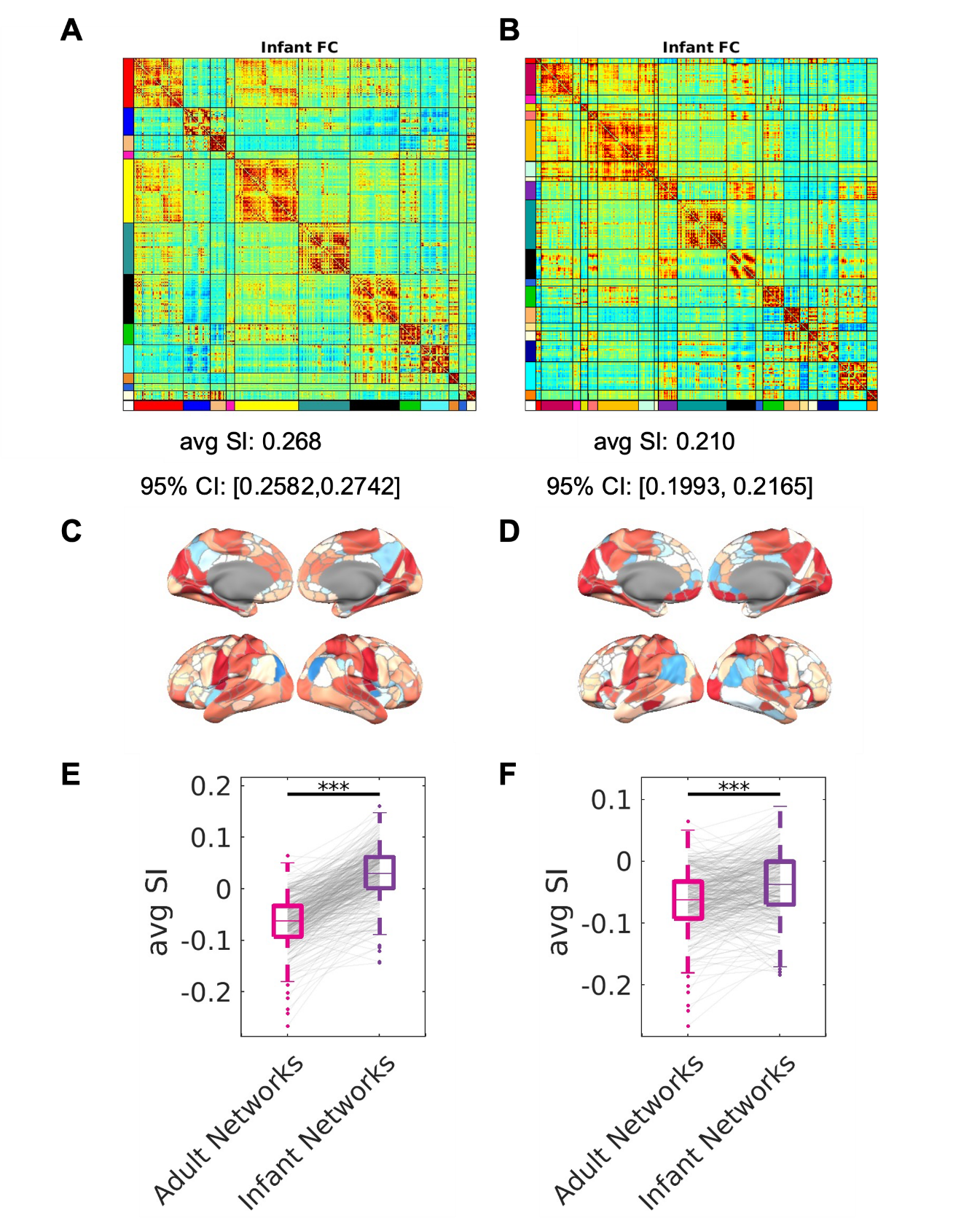


**Supplementary Figure 6.** *Infant FC sorted by the Tu (326) 12 and 19 networks.* A) The average FC for 313 BCP sessions sorted by Tu (326) 12 networks. B) The average FC for 313 BCP sessions sorted by Tu (326) 19 networks. C-D) Silhouette index for each area parcel for A-B. E-F) Average silhouette index for individual sessions. *** *p* < 0.001.


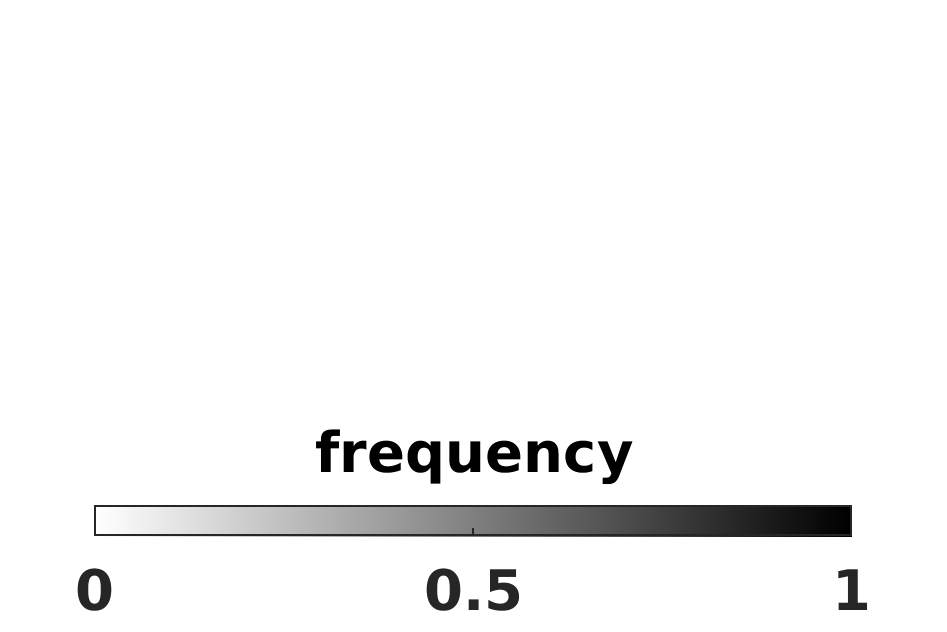

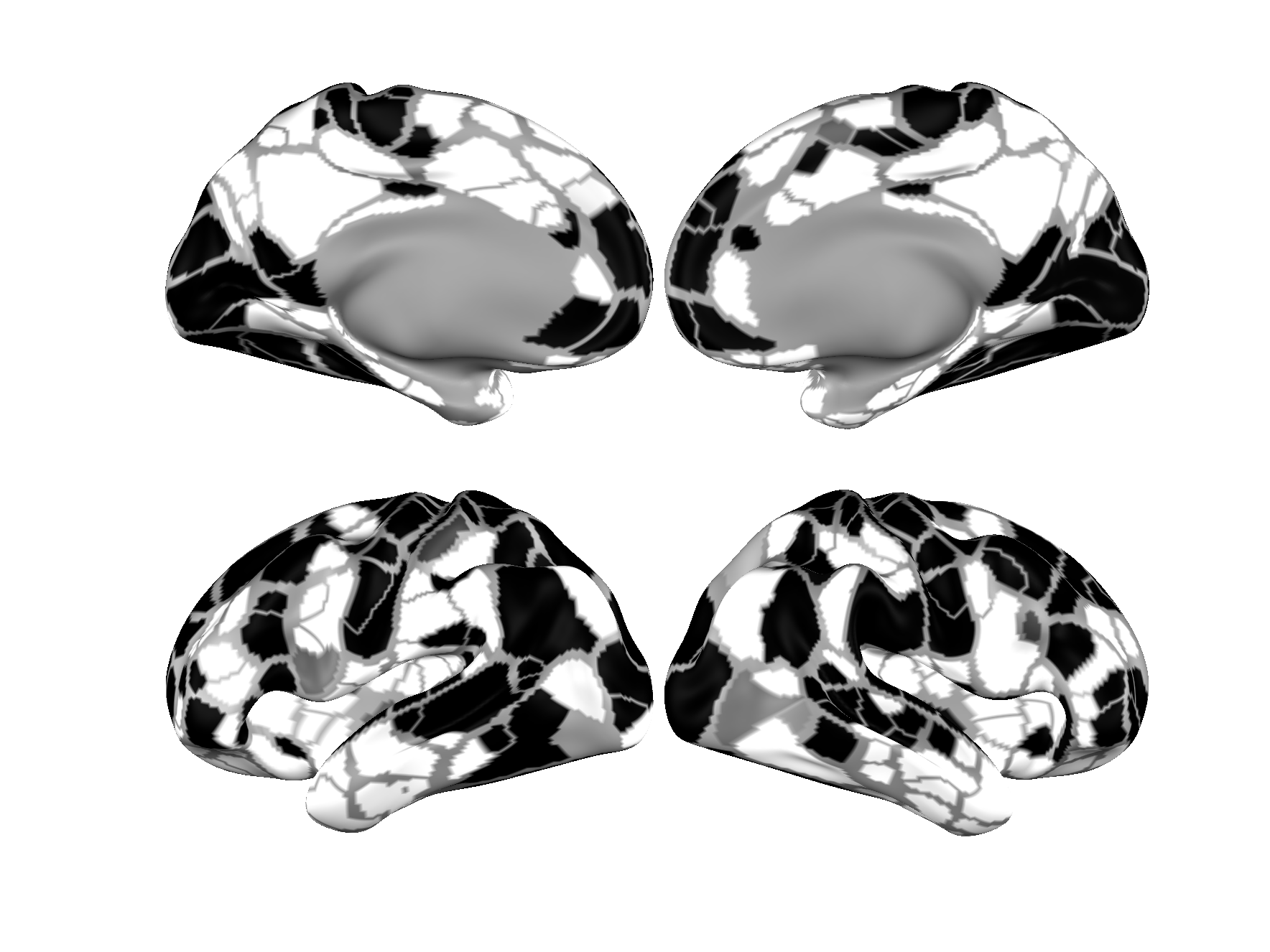


**Supplementary Figure 7.** *Frequency of SI > 0 across 1000 bootstraps.*


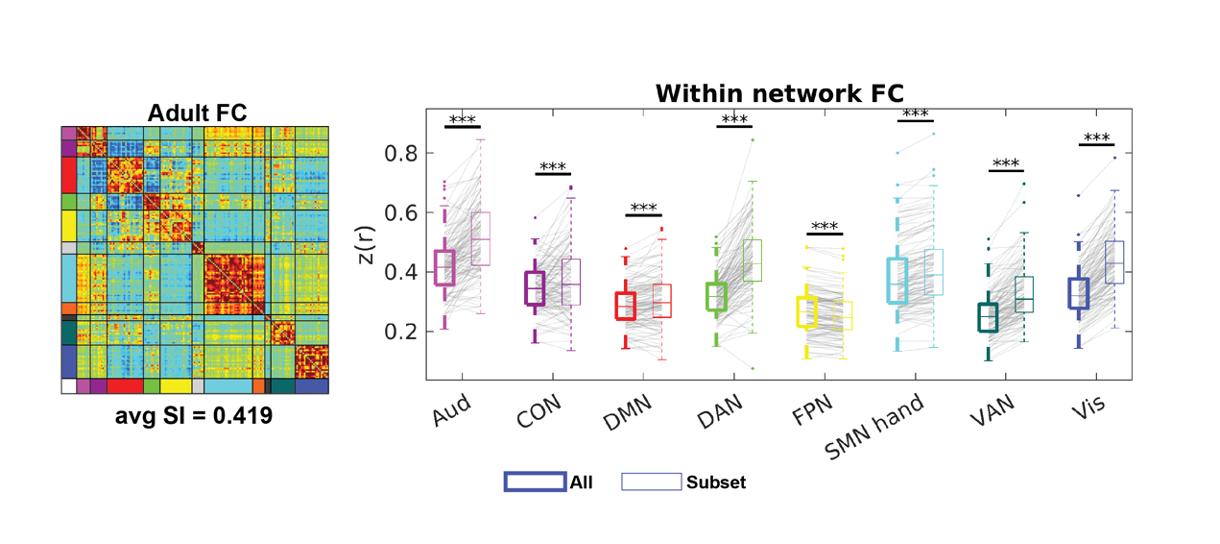


**B**

**Supplementary Figure 8.** *Adult FC using our area subset.* A) The sorted average FC in adults with our area subset. B) The average within-network FC with all areas (left) versus our area subset subset (right) across sessions. *** *p* < 0.001.

**A**


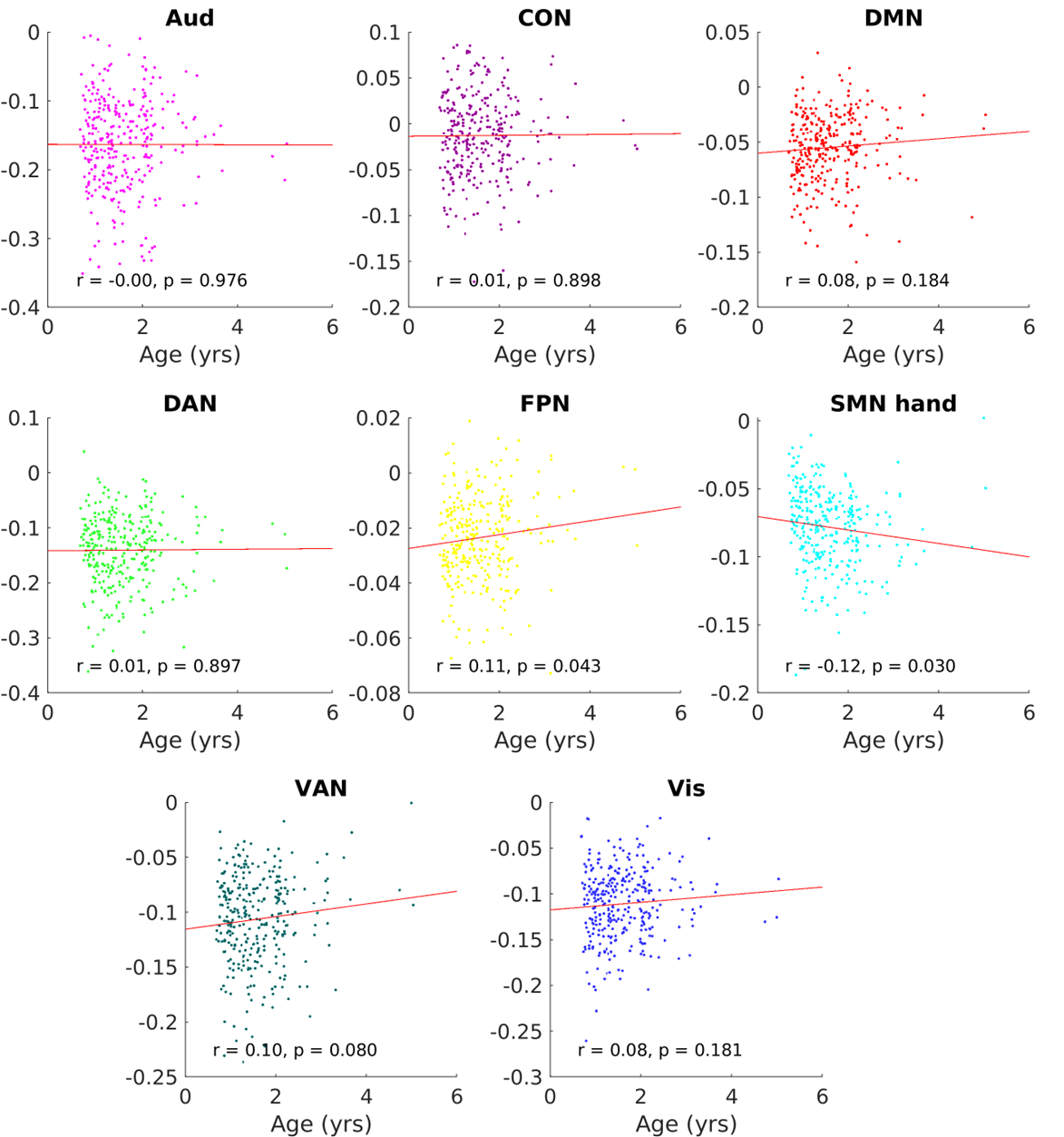


**Supplementary Figure 9.** *Within-network FC difference (All – Subset) across eight partially-retained networks.*


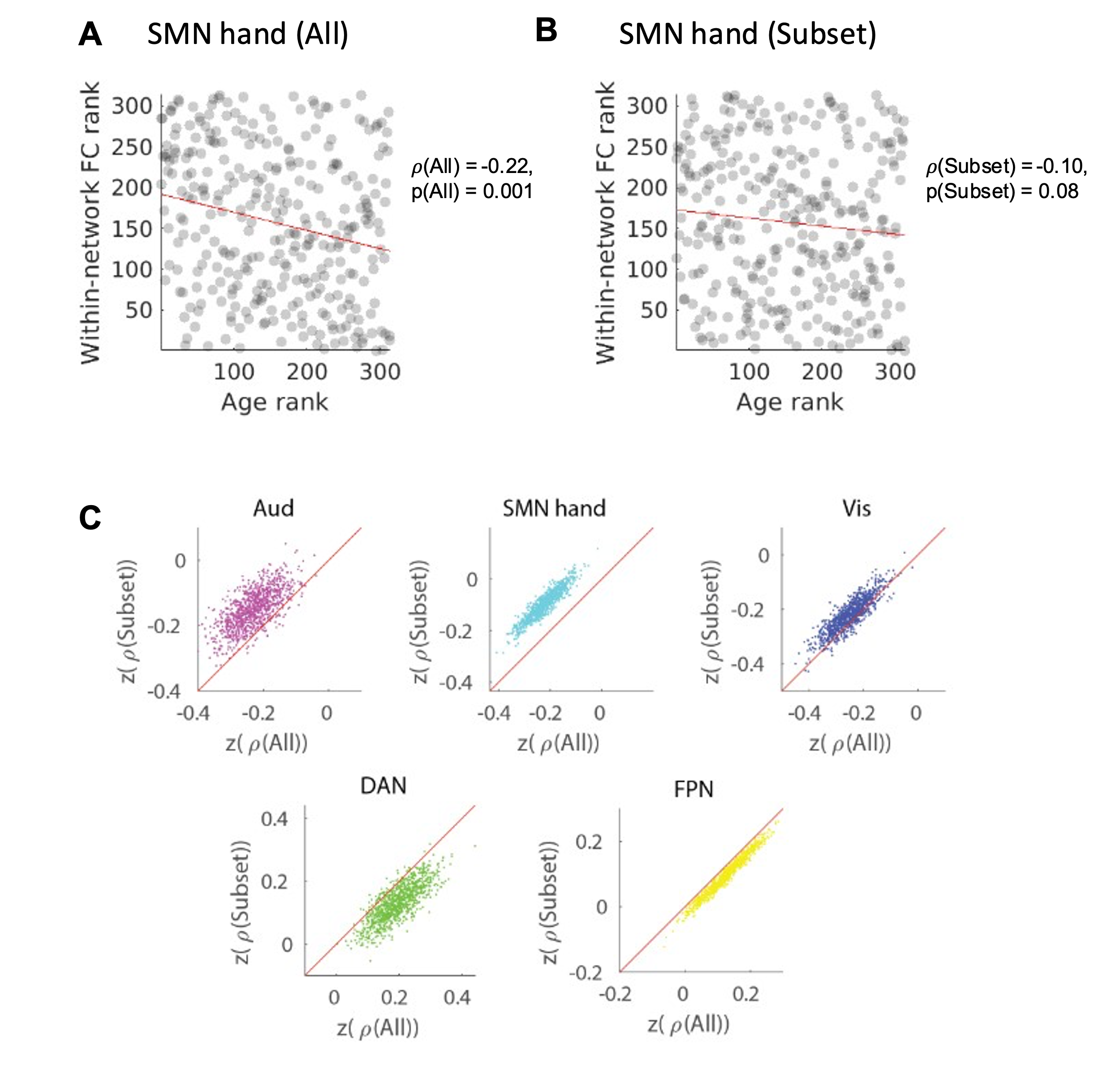


**Supplementary Figure 10.** *Correlation between age and within-network FC in using our area subset versus all areas.* A) Scatter plot of within-network FC versus age for SMN hand network using all areas. B) Scatter plot of within-network FC versus age for SMN hand network using our area subset. C) The within-network FC for three networks is negatively correlated with age (Aud, SMN hand, Vis), and the within-network FC for two networks is positively correlated with age (DAN, FPN). The x-axis is the Fisher-Z-transformed Spearman’s correlation (*ρ*) between within-network FC using all areas and age. The y-axis is the Fisher-Z-transformed Spearman’s correlation (*ρ*) within-network FC using our area subset and age). Each data point represents a bootstrap sample of sessions (N = 1000). Red line shows the line of least-squared fit in A-B and the line of identity in C.


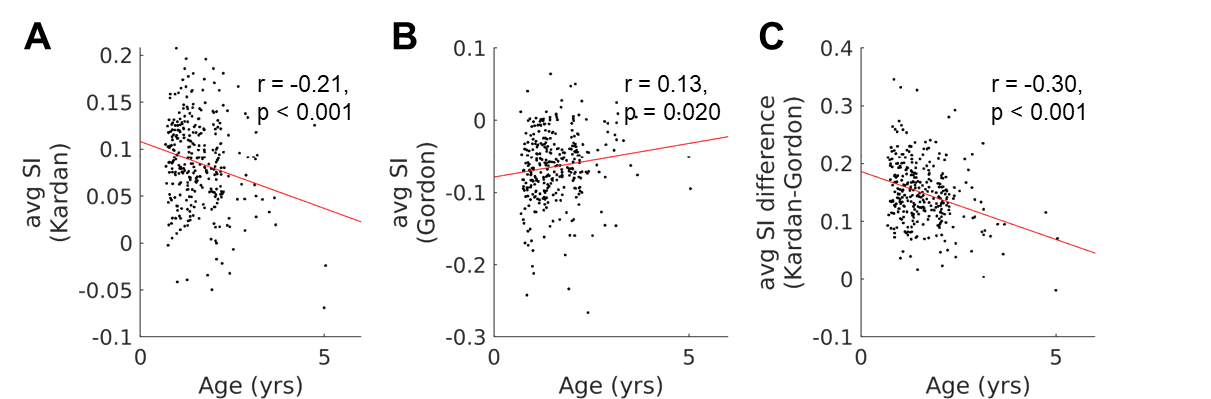


**Supplementary Figure 11.** *A scatter plot between chronological age in years and average SI for individual BCP sessions.* A) infant networks (“Kardan”). B) adult networks (“Gordon”). C) Difference in infant networks (“Kardan”) and adult networks (“Gordon”).


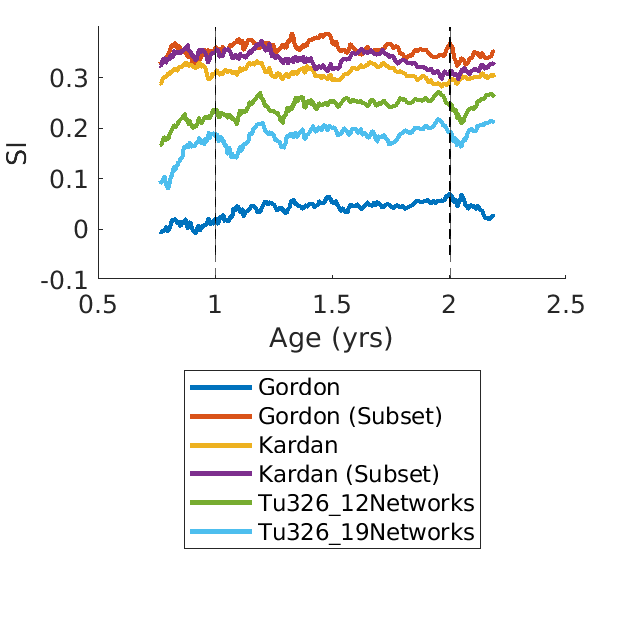


**Supplementary Figure 12.** *Moving average analysis (Figure 4A) adding the results using infant networks from an independent dataset.*


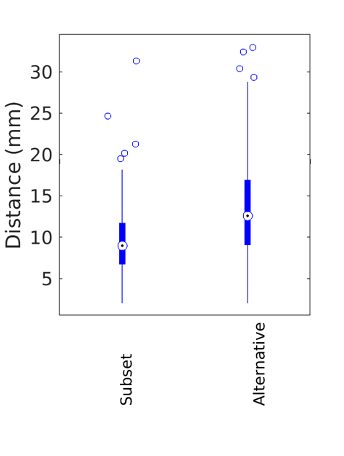


**Supplementary Figure 13.** *Distance between our area subset and alternative areas to the 153 Dworestky high consensus ROI.*


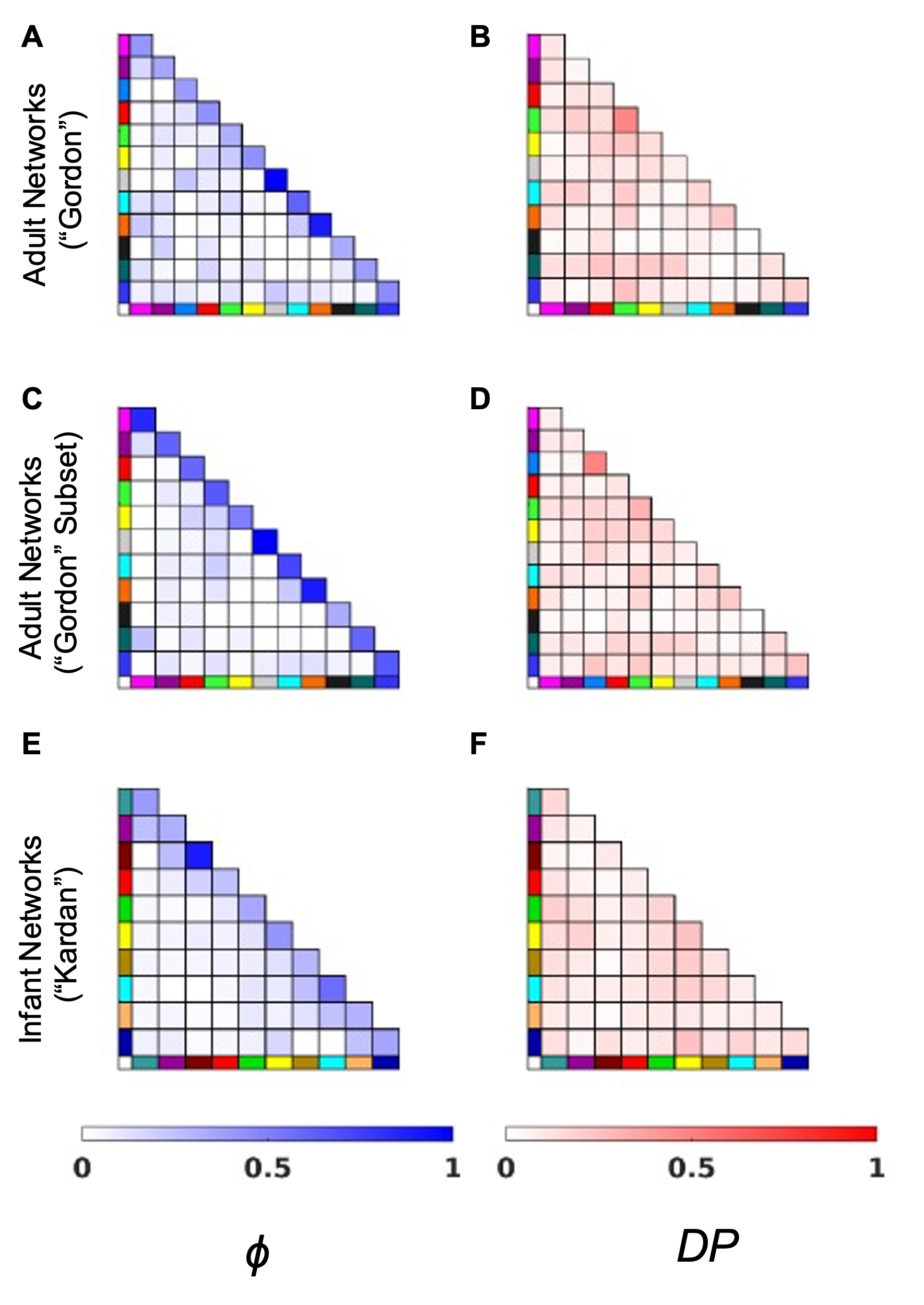


**Supplementary Figure 14.** *Fraction of high consistency (ϕ) and high differential power (DP) edges (top 10%) across (A-B) adult networks (“Gordon”), (C-D) adult networks (“Gordon” Subset), (E-F) infant networks (“Kardan”).*


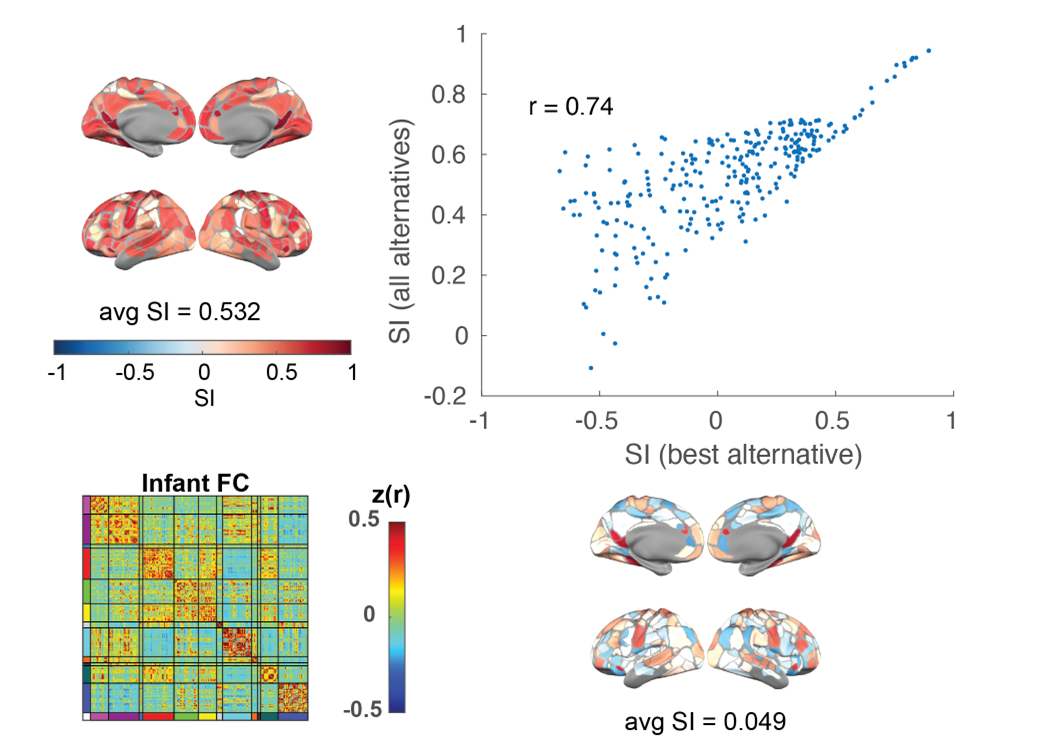


**Supplementary Figure 15.** *Correlation between silhouette index calculated with the best network or with all alternative networks*.

| **Supplementary Table 1** | | | |
| --- | --- | --- | --- |
|  | Cohort | BCP | WashU 120 |
| **Acquisition** | Location | University of Minnesota | Washington University in St. Louis |
|  | Scanners | Siemens Prisma 3T Scanner | Siemens 3T Trio Tim |
|  | Headcoil | 32-channel | 12-channel |
|  | Sequence type | Gradient-echo EPI | Gradient-echo EPI |
|  | Resolution (BOLD) | 2mm isotropic | 4 mm isotropic |
|  | Phase encoding direction | AP+PA | AP |
|  | TR (s) | UMN - 0.72 (N = 70), 0.8 (N = 107) | 2.5 |
|  | TE (ms) | 37 | 27 |
|  | Resolution (T1) | 0.8 mm | 1 mm |
|  | Multi-band factor | 8 | N/A |
|  | State | Natural Sleep | Fixation on crosshair |
|  | Average clean data frames |  | 14.0 min |
|  | Frames per BOLD run | 420 | 120 |
|  | BOLD runs | 2-4 | 2 |
| **Processing** | Processing pipeline version | DCAN-Infant (0.0.22) | adult EPI (BOLD) preprocessing pipeline using the 4dfp tool suite |
|  | Distortion correction | ANTs SyN registration | None |
|  | Bias field correction | N4 method | None |
|  | Denoising | Respiratory notch filter, demean, detrend, 24 parameters nuisance regression, remove FD>0.3mm to apply bandpass filtering (0.008-0.09 Hz) then interpolate the missing frames | Demeaning and detrending, , multiple regression including: whole-brain, ventricular and white matter signals, and motion regressors derived by Volterra expansion (Friston et al. 1996), and a band-pass filter (0.009 Hz < f < 0.08Hz). |
|  | Scrubbing threshold | filtered FD<0.2mm, outlier (across-vertex STD on low FD frames >3MAD of the median of all frames) | FD<0.2mm, at least 5 consecutive low-motion frames |
|  | Tissue Segmentation | ANTs Joint Label Fusion was performed using a set of ALBERT atlases with segmentations generated and manually corrected by DCAN for ages 0-5 months old. For older ages, the atlases used for JLF were a set of 10 ABCD subjects for which we generated segmentations. Manual curation of tissue segmentation was performed where necessary. | FreeSurfer’s default recon-all processing pipeline (version 5.0) |
|  | Surface reconstruction & registration | Modified FreeSurfer reconstruction (no hires, aseg from JLF, adjusted class means of tissue to fit T1w contrasts), registered to fs_LR32k using spherical registration (with MSMsulc). | FreeSurfer’s default recon-all processing pipeline (version 5.0) |
|  | BOLD data geodesic smoothing | σ = 2.55 mm | σ = 2.55 mm |
| **Quality Control** |  |  |  |
|  |  | BrainSwipes crowdsource ratings with average aggregated passing rate >75% for anatomical or functional images + manual screening. | N/A |
|  | Data availability | <https://nda.nih.gov/edit_collection.html?id=2848> | <https://legacy.openfmri.org/dataset/ds000243/> |

| **Supplementary Table 2** | | |
| --- | --- | --- |
| x | y | z |
| -18.8 | -48.7 | 65 |
| -51.8 | -7.8 | 38.5 |
| -18.4 | -85.5 | 21.6 |
| -47.2 | -58 | 30.8 |
| -38.1 | 48.8 | 10.5 |
| -55.9 | -47.7 | -9.3 |
| -14.4 | -57.8 | 18.4 |
| -8.8 | -49.8 | 4.2 |
| -11.3 | -83.2 | 3.9 |
| -1.7 | -17.7 | 39.1 |
| -10 | 33.9 | 21.5 |
| -10.7 | -47.5 | 60.3 |
| -15.6 | -33.1 | 66.1 |
| -10.9 | -29.3 | 69.5 |
| -6.6 | -20.4 | 74.2 |
| -10.8 | -41.1 | 64.9 |
| -5 | -28.2 | 60.4 |
| -5.4 | -15.9 | 48.8 |
| -35.8 | -29.7 | 54.5 |
| -41.5 | -12.5 | 50.4 |
| -42.1 | -4.5 | 47.3 |
| -27.3 | 1.9 | 52.9 |
| -19.8 | 6.4 | 55.7 |
| -19.5 | 30.1 | 45.5 |
| -36.8 | -22.8 | 61.9 |
| -20.5 | -24.9 | 64.5 |
| -23.4 | -13.8 | 64.2 |
| -17.2 | -8.6 | 67.9 |
| -28.6 | -44.7 | 61.7 |
| -31.1 | -48.9 | 47.1 |
| -42.9 | -45 | 43 |
| -51.5 | -11.9 | 29.7 |
| -51.7 | -30.9 | 39.9 |
| -27.5 | -37.2 | 61.4 |
| -47.2 | -31.4 | 54.8 |
| -46.1 | -17.8 | 52.7 |
| -44.8 | -54 | 14.6 |
| -51.6 | -55.9 | 11.4 |
| -48.1 | -40 | 2.4 |
| -46.3 | -41.4 | 25.9 |
| -52.7 | -20.6 | 5.4 |
| -58.7 | -29.9 | 11.1 |
| -40.6 | -38.3 | 14.5 |
| -38.7 | -16 | -5.3 |
| -50 | 20.8 | 10.6 |
| -37.7 | 2.9 | 11.7 |
| -40.3 | 50.4 | -4.8 |
| -32.5 | 17.2 | -7.8 |
| -44.3 | 33.2 | -7.2 |
| -45.4 | 28.8 | 0.8 |
| -20.4 | -64.6 | 51.4 |
| -34.1 | -61 | 42.4 |
| -31.3 | -84.2 | 9 |
| -34.2 | -86.6 | -0.5 |
| -46.2 | -57.7 | -7.9 |
| -55.1 | -32.3 | 23 |
| -43 | 19.4 | 33.5 |
| -40.2 | 23.6 | 23.3 |
| -48.6 | 7.5 | 11.1 |
| -5.9 | 54.8 | -11.3 |
| -6.8 | 38.2 | -9.4 |
| -33.8 | -33.2 | -15.4 |
| -28.8 | -58.8 | -9.1 |
| -34.4 | -63.9 | -15.7 |
| -34.3 | -43.8 | -21.6 |
| -5.4 | -88 | 18.6 |
| -8.6 | -77.5 | -3.5 |
| -22.6 | -81.7 | -11.7 |
| -22.5 | -37.1 | -15 |
| -15.9 | 48.6 | 37.2 |
| -19.5 | 56.3 | 27.5 |
| -21.3 | 63.1 | 1.9 |
| -28.6 | 50.9 | 10.1 |
| -6.5 | 54.7 | 18.1 |
| -15.7 | 64.7 | 13.7 |
| -26.2 | 26.6 | 38.8 |
| -29.3 | 16.8 | 50.7 |
| -41.7 | 16.1 | 47.5 |
| -54.4 | -1.4 | -0.7 |
| -59 | -18 | -3 |
| 20.8 | -48.2 | 66.1 |
| 49.6 | -7.4 | 36.1 |
| 22 | -84.6 | 23.7 |
| 47.9 | -42.5 | 41.5 |
| 38.1 | 45.9 | 7.7 |
| 59.7 | -41 | -10.9 |
| 13.8 | -54.1 | 10.9 |
| 15.5 | -74.1 | 9.4 |
| 6.7 | 5 | 55.9 |
| 8.4 | 34.7 | 22.6 |
| 3 | -19.6 | 37.9 |
| 8.8 | 10.8 | 45.9 |
| 16.5 | -32.8 | 67.7 |
| 4.8 | -27.1 | 64.8 |
| 11.9 | -40.7 | 67 |
| 5.1 | -17.1 | 51.6 |
| 6.8 | -8.1 | 50.9 |
| 42.3 | -11 | 47.3 |
| 42.5 | -2.3 | 47.2 |
| 29.2 | 1.9 | 52.4 |
| 21.9 | 21 | 46.2 |
| 38.1 | -22.4 | 60.3 |
| 19.7 | -25 | 65.2 |
| 12.4 | -28.3 | 69.6 |
| 29.2 | -13.5 | 64.2 |
| 17 | -16.9 | 70.9 |
| 20.9 | -6.4 | 65 |
| 29.5 | -42.5 | 60.4 |
| 38.8 | -42.6 | 40.4 |
| 53.9 | -8.3 | 26.1 |
| 28 | -34.8 | 63.1 |
| 39.2 | -34.6 | 57.5 |
| 37.3 | -25.9 | 50.9 |
| 47.8 | -15.1 | 49.3 |
| 48.9 | -53 | 28.6 |
| 57.5 | -45.3 | 9 |
| 60.9 | -38.7 | 1.7 |
| 54.9 | -27 | 29.6 |
| 57.1 | -17 | -2.6 |
| 53.8 | -15.8 | 5.2 |
| 47.4 | -39.6 | 13.2 |
| 45.5 | -37.3 | 3.4 |
| 48.5 | -26.5 | -0.1 |
| 60 | -25.2 | 10.2 |
| 38.8 | -14.4 | -5 |
| 42.8 | 48.3 | -5.1 |
| 45.2 | 30.7 | -5.6 |
| 30.6 | 22.8 | -4.7 |
| 7.7 | -85.6 | 31.6 |
| 35.4 | -77.1 | 21.1 |
| 41.5 | -53.5 | 44 |
| 33.5 | -48.2 | 49.4 |
| 31.7 | -85.7 | 2.4 |
| 43.8 | -67.2 | 2 |
| 57 | -53.8 | -1.1 |
| 37.8 | 28.7 | 35.6 |
| 41.8 | 29.1 | 21.6 |
| 50.1 | 3 | 3.9 |
| 38.6 | 18.8 | 25.5 |
| 28.4 | 57 | -5.1 |
| 4.8 | 65.1 | -7.1 |
| 7.2 | 48.4 | -10.1 |
| 34.6 | -35.6 | -12.3 |
| 34.6 | -23.9 | -20.4 |
| 26.9 | -69.1 | -6.6 |
| 34.9 | -44 | -20 |
| 13.8 | -92.3 | 14.7 |
| 10.5 | -73.8 | -1.5 |
| 20.4 | -87.3 | -6.6 |
| 5.1 | -80.2 | 23.1 |
| 24.5 | -36.2 | -13.2 |
| 21 | 32.8 | 42.1 |
| 21.4 | 42.8 | 35.1 |
| 23.5 | 59.1 | 4.9 |
| 30.9 | 52.2 | 9.9 |
| 8.2 | 53.8 | 14 |
| 5.9 | 54.9 | 29.4 |
| 13.8 | 46.7 | 42.1 |
| 6.8 | 44.5 | 34.8 |
| 30.6 | 18.9 | 48.7 |
| 42.4 | 19.5 | 48.2 |
| 38.9 | 9.6 | 42.7 |
| 39.7 | -22.5 | 2.6 |
| 55.8 | 2 | -2 |
| 57.1 | -6.3 | -7.7 |
| 46.6 | -21.5 | -8.5 |

|  | **Gordon All** | **Gordon Subset** | **Kardan** |
| --- | --- | --- | --- |
| within-network | 44.1% | 63.1% | 49.5% |
| between-network | 8.3% | 6.5% | 5.4% |

**Supplementary Table 3.** Percentage of highly consistent (*φ*) edges across different network assignment schemes

| **within-network** | **Gordon All** | **Gordon Subset** |
| --- | --- | --- |
| **Aud*** | 41.30% | 84.21% |
| **CON*** | 35.64% | 61.73% |
| PMN | 40.00% | / |
| **DMN*** | 43.17% | 59.07% |
| **DAN*** | 31.45% | 65.17% |
| **FPN*** | 43.12% | 50.84% |
| RTN | 100% | 100% |
| **SMN hand*** | 61.45% | 72.82% |
| SMN mouth | 89.29% | 89.29% |
| Sal | 33.33% | 33.33% |
| **VAN*** | 38.34% | 60.94% |
| **Vis*** | 45.89% | 65.17% |

**Supplementary Table 4.** Percentage of highly consistent (*φ*) edges across different network assignment schemes. The eight partially retained networks were bolded and had an asterisk.

|  | **Gordon All** | **Gordon Subset** | **Kardan** |
| --- | --- | --- | --- |
| within-network | 16.50% | 14.73% | 12.79% |
| between-network | 10.81% | 10.98% | 9.93% |

**Supplementary Table 5.** Percentage of high differential power (*DP*) edges across different network assignment schemes.

| **within-network** | **Gordon All** | **Gordon Subset** |
| --- | --- | --- |
| **Aud*** | 7.97% | 10.53% |
| **CON*** | 8.33% | 4.94% |
| PMN | 50.00% | / |
| **DMN*** | 10.73% | 11.59% |
| **DAN*** | 30.24% | 47.19% |
| **FPN*** | 15.58% | 13.91% |
| RTN | 7.14% | 7.14% |
| **SMN hand*** | 17.92% | 15.82% |
| SMN mouth | 21.43% | 21.43% |
| Sal | 0% | 0% |
| **VAN*** | 14.62% | 12.02% |
| **Vis*** | 24.97% | 18.43% |

**Supplementary Table 6.** Percentage of high differential power (*DP*) edges across different network assignment schemes. The eight partially retained networks were bolded and had an asterisk.
